# Epididymal Dissociation to Facilitate Vasectomy Reversal in a Patient With Sizeable Vasal Defect: A Case Report

**DOI:** 10.1155/criu/7984429

**Published:** 2025-10-29

**Authors:** Jack C. Millot, Aaron J. Smith, Scott D. Lundy

**Affiliations:** ^1^Case Western Reserve University School of Medicine, Cleveland, Ohio, USA; ^2^Glickman Urological and Kidney Institute, Cleveland Clinic, Cleveland, Ohio, USA; ^3^Northwell Health Urology, Northwell Health, Great Neck, New York, USA

## Abstract

**Background:**

Close to a quarter of men seek paternity after vasectomy. Microsurgical vasectomy reversal is a common choice for men seeking children after vasectomy, with outcomes dependent on surgical expertise and intraoperative decision-making. Here, we describe the case of a patient with an unexpectedly long vasal gap that necessitated the dissection of the tail and midbody of the epididymis from the testis during a vasoepididymostomy.

**Case Discussion:**

We report a case of a 31-year-old male who underwent vasectomy reversal that required a rare surgical approach. During the operation, we discovered a secondary distal obstruction and an additional proximal obstruction on the patient's left side, which resulted in a long vasal gap that did not permit a tension-free vasoepididymostomy. To bridge the long gap, the tail and midbody of the epididymis were carefully dissected and mobilized from the testis. Dissociation of the epididymis from the testis allowed a tension-free vasoepididymostomy. Postoperative semen analysis confirmed patency, and the patient had no concerns.

**Conclusion:**

Dissection of the tail and midbody of the epididymis is discussed in textbooks, but is not readily documented in real-world patients. Here, we provide a case discussion where the dissection of the epididymis from the testis was successfully performed to gain additional length to bridge a long vasal gap.

## 1. Introduction

Over 500,000 vasectomies are performed annually in the United States [1]. About 20% of men who undergo vasectomy will express a desire for children after the procedure for a variety of reasons (e.g., new partner, changes in financial or religious situations, and unexpected desire for an additional child with a stable partner) [2]. Options for men who seek postvasectomy paternity include surgical reversal (vasovasostomy or vasoepididymostomy) or surgical sperm retrieval and assisted reproductive technology (ART). Vasectomy reversal is the only option that allows for natural conception and tends to be more cost-effective if ART insurance coverage is not available [3]. In the United States, approximately 2%–6% of men with a vasectomy will undergo vasectomy reversal [4].

Successful vasectomy reversal is predicated on microsurgical expertise and intraoperative decision-making of the surgeon to achieve a watertight and tension-free anastomosis [5]. In patients with long vasal gaps or who require a vasoepididymostomy due to a proximal obstruction, this can be challenging. In this case report, we describe a patient with an unexpectedly long vasal gap due to a distal secondary vasal obstruction and dry proximal vas concerning a third proximal obstruction for which a vasoepididymostomy was indicated. Due to inadequate vasal length, we report an uncommon technique to dissociate the tail and midbody of the epididymis from the testicle to gain sufficient length to perform a vasoepididymostomy. This technique is theorized in the literature, but, to our knowledge, has not been reported clinically.

## 2. Case Presentation

A 31-year-old male with no desire for children underwent a vasectomy and 5 years later presented to our department seeking fertility restoration. He reported a remote history significant for left epididymitis requiring antibiotic therapy. On physical exam, his testes were 18 cc with palpable, nontender, full epididymides bilaterally. The exam was remarkable for a difficult-to-palpate defect on the right and a left defect in the high scrotum.

**Figure 1:**
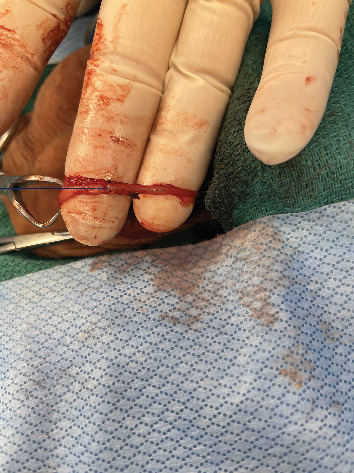
Obstructed segment of vas deferens located approximately 2 cm from the distal obliterated segment.

**Figure 2:**
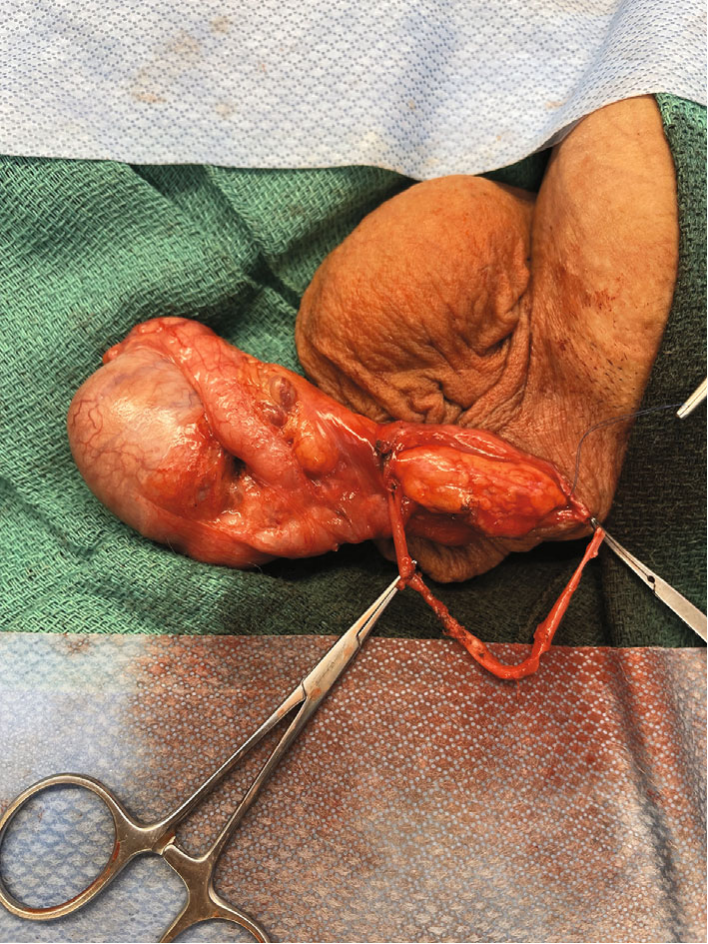
Large vasal gap (approximately 8 cm) from secondary obstruction of abdominal vas deferens.

**Figure 3:**
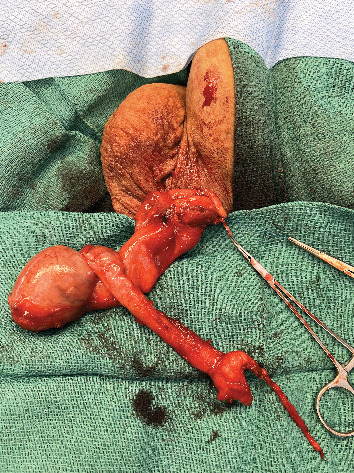
Epididymal tail and midbody dissected from the testicle and rotated cranially.

**Figure 4:**
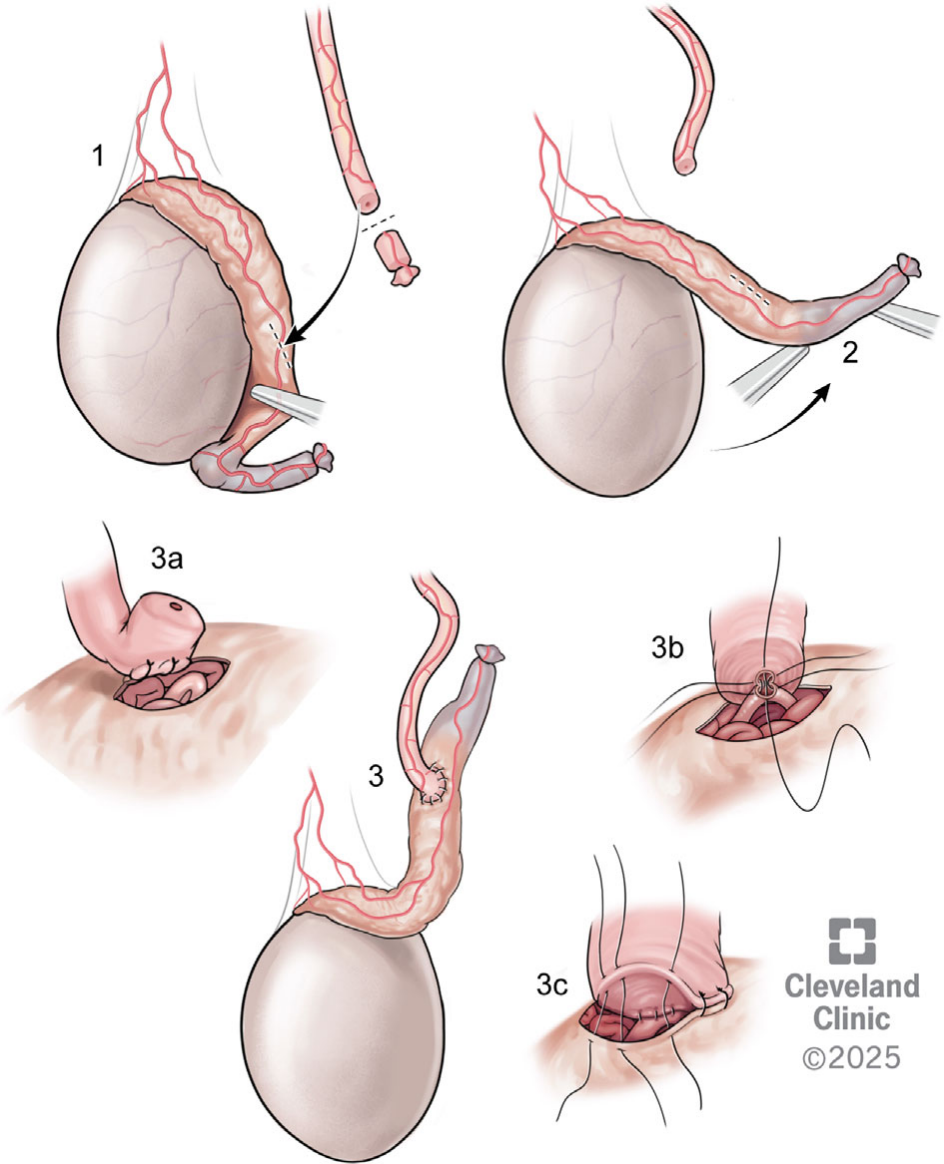
Application of epididymal dissection and rotation for longitudinal intussusception vasoepididymostomy. (1) Dissection of epididymal tail and midbody from the testicle. (2) Rotation of the dissected epididymal segment cranially. (3a) Vas deferens secured to the tunica albuginea with 9-0 nylon suture. (3b) Intussusception of the epididymal tubule into the vasal segment. (3c) Closure of the site with the tunica albuginea secured to the vasal adventitia.

After a discussion of the various options available for fertility restoration, the patient elected to proceed with microsurgical vasectomy reversal with cryopreservation. Under general anesthesia, a 1 cm vertical incision was made over the easily palpable left vasal defect, the proximal and distal vasal segments were mobilized, and sufficient length was obtained to perform a repair. The distal aspect was incised sharply, and a 2-0 prolene suture was passed through the lumen, but an unexpected additional distal obliterated segment was encountered 2 cm above the vasal defect (Figure 1). The subsequent excision of the distal defect was followed by retesting for patency with 2-0 prolene suture. In total, after the patency of the distal vasal segment was obtained, the resulting vasal defect measured approximately 8 cm (Figure 2). Incising the testicular vasal stump returned thick pasty fluid devoid of sperm or sperm parts. The testicular vas was lavaged with saline several times, but no sperm could be identified. Subsequently, the incision was extended, and the testicle was delivered. Upon incising the tunica vaginalis, an assessment of the epididymis revealed dilated tubules in the head and body of the epididymis but flat tubules in the tail, indicative of epididymal obstruction. The decision to perform a vasoepididymostomy was made, and additional length was obtained from the abdominal vas to the level of the inguinal canal. Given the unexpectedly long defect, however, a tension-free anastomosis could not be performed without further maneuvers. The tail of the epididymis was then carefully and sharply mobilized from the lower pole of the testicle. Great care was taken to avoid injury to the epididymal and testicular blood supply, which was identified using a micro-Doppler. Pinpoint bipolar cautery was used judiciously. The dissection was carried up to the level of the testicular hilum, at which point an additional 3 cm of length had been obtained by rotating the epididymis cranially (Figure 3). An additional Doppler interrogation of the testicle revealed excellent blood flow and no concern for vascular compromise. The left vasoepididymostomy was then performed using the longitudinal intussusception vasoepididymostomy (LIVE) technique (Figure 4). Briefly, the tunica overlying the epididymal tubules was carefully incised, and individual tubules were isolated. A candidate tubule was selected, and the vas deferens was brought in close approximation using a series of 9-0 sutures. Two double-armed 10-0 sutures were placed longitudinally in the epididymal tubule, and the tubule was incised, revealing copious milky fluid with copious sperm upon examination under the benchtop microscope. The sutures were then passed through the vasal wall and tied to intussuscept the tubule into the vasal lumen. The tunical defect was then closed circumferentially around the vas deferens, completing the anastomosis. The testicle was returned to the scrotum. The right side was then assessed and noted to have a short vasal defect, but inspection of the testicular vasal lumen revealed no fluid despite lavage. A traditional vasoepididymostomy was then performed on the right side without difficulty. The superficial layers were closed using 3-0 vicryl, and the skin was closed using 4-0 vicryl and surgical glue.

The patient had an uneventful postoperative recovery with minimal pain controlled with acetaminophen and ibuprofen. After the procedure, the patient was doing well, with no concerns at his 1-month follow-up exam. Semen analysis performed at 4 months showed a volume of 7.4 mL, a concentration of 13 M/mL, 23% motility, and a total motile sperm count of 22.1 million. Repeat semen analysis performed at 8 months postoperation resulted in a volume of 4.5 mL, a concentration of 14.5 million/mL, 28% motility, and a total motile sperm count of 18.27 million. He and his partner are currently actively trying to conceive.

## 3. Discussion

Maintaining a watertight and tension-free anastomosis is a fundamental surgical principle critical to successfully restoring vasal patency, preventing immune response to haploid sperm, and ultimately facilitating biological fatherhood following vasectomy [2, 5, 6]. In the vast majority of cases, the testicle and scrotum provide sufficient mobility to achieve this without further maneuvers, even when the vasal defect is longer than expected. This case report describes the successful utilization of the epididymis itself as a rotational tissue graft to recover lost vasal length, a technique proposed previously but not reported to our knowledge.

In cases with large vasal gaps, traditionally, the first maneuver consists of mobilizing the abdominal vas [2, 7, 8]. The other straightforward approach to gaining vasal remnant length is to dissect and mobilize the convoluted vas from the epididymal tunic [2, 7, 9]. If the surgeon cannot achieve a tension-free anastomosis despite mobilizing the abdominal and testicular segments, an orchidopexy can be used to position the testis higher in the scrotum, with an altered orientation (horizontal or upside down) if needed [6, 7, 9]. When these are insufficient, others have advocated for abdominal vas mobilization and rerouting in an ectopic medial position external to the inguinal canal [3, 6, 7].

To our knowledge, the technique described here (epididymal dissociation and rotation) has not been previously described in a real-world setting. The dissection of the tail and body of the epididymis is reliant on the maintenance of the superior epididymal artery, which derives from the testicular artery. Importantly, the superior epididymal artery, along with the extensive anastomotic network along the epididymis, can provide blood supply to the entirety of the epididymis even in the absence of contributions from the deferential artery via the inferior epididymal artery [7, 8]. It should be noted that the inferior epididymal artery is supplied by the deferential artery, which is ligated during vasectomy.

In the case presented here, the high abdominal vas defect, coupled with secondary abdominal vasal obstruction and the suspected epididymal obstruction, left limited potential for obtaining additional length via the abdominal vas. While abdominal rerouting of the vas deferens may have succeeded, this is an invasive approach with the potential for visceral injury and with a more substantial recovery period and has not been discussed with the patient preoperatively.

Previously, it has been suggested that if vasoepididymostomy is not feasible due to the vasal gap, then a vasovasostomy should be performed, understanding that this may ultimately not be successful [2]. Given our experience in this case, however, we suggest considering epididymal dissociation and rotation if a vasoepididymostomy is indeed clinically indicated.

One of the limitations of the case presented here is the unilateral application of this approach in our patient and an inability to confirm bilateral patency. Although his semen analysis results at 4 months were indeed promising, we cannot exclude isolated contralateral patency. It is also important to note that in cases where vasoepididymostomy to the head of the epididymis is required, this maneuver fails to provide additional length beyond what could be obtained from orchidopexy alone.

## 4. Conclusion

We report a case of a vasoepididymostomy where we dissected the tail and body of the epididymis from the testis to bridge a considerable vasal gap that resulted in patency on semen analysis. Provided the lack of previously published patient experience with performing tail and midbody epididymal dissection, we find that this case reassures the application of this approach in rare instances.

## Data Availability

The data that support the findings of this study are available on request from the corresponding author. The data are not publicly available due to privacy or ethical restrictions.

## Nomenclature

ART: assisted reproductive technology
LIVE: longitudinal intussusception vasoepididymostomy

## References

[1] Ostrowski K. A., Holt S. K., Haynes B., Davies B. J., Fuchs E. F., Walsh T. J. (2018). Evaluation of Vasectomy Trends in the United States. *Urology*.

[2] Fantus R. J., Halpern J. A. (2021). Vasovasostomy and Vasoepididymostomy: Indications, Operative Technique, and Outcomes. *Fertility and Sterility*.

[3] Schwarzer J. U., Steinfatt H. (2013). Current Status of Vasectomy Reversal. *Nature Reviews Urology*.

[4] Kirby E. W., Hockenberry M., Lipshultz L. I. (2017). Vasectomy Reversal: Decision Making and Technical Innovations. *Translational Andrology and Urology*.

[5] Andino J. J., Gonzalez D. C., Dupree J. M., Marks S., Ramasamy R. (2021). Challenges in Completing a Successful Vasectomy Reversal. *Andrologia*.

[6] Patel A. P., Smith R. P. (2016). Vasectomy Reversal: A Clinical Update. *Asian Journal of Andrology*.

[7] Chan P. T. (2013). The Evolution and Refinement of Vasoepididymostomy Techniques. *Asian Journal of Andrology*.

[8] Lyu K. L., Zhuang J. T., Li P. S. (2018). A Novel Experience of Deferential Vessel-Sparing Microsurgical Vasoepididymostomy. *Asian Journal of Andrology*.

[9] Hayden R. P., Li P. S., Goldstein M. (2019). Microsurgical Vasectomy Reversal: Contemporary Techniques, Intraoperative Decision Making, and Surgical Training for the Next Generation. *Fertility and Sterility*.

